# Inverse Relationship between Serum 25-Hydroxyvitamin D and Elevated Intraocular Pressure

**DOI:** 10.3390/nu15020423

**Published:** 2023-01-13

**Authors:** Jun-Hyuk Lee, Yu-Jin Kwon, Hye Sun Lee, Jee Hye Han, Boyoung Joung, Sung Jin Kim

**Affiliations:** 1 Department of Family Medicine, Nowon Eulji Medical Center, Eulji University School of Medicine, Seoul 01830, Republic of Korea; 2 Department of Medicine, Graduate School of Hanyang University, Seoul 04763, Republic of Korea; 3 Department of Family Medicine, Yongin Severance Hospital, Yonsei University College of Medicine, Seoul 16995, Republic of Korea; 4 Biostatistics Collaboration Unit, Department of Research Affairs, Yonsei University College of Medicine, Seoul 06273, Republic of Korea; 5 Division of Cardiology, Department of Internal Medicine, Yonsei University College of Medicine, Seoul 03722, Republic of Korea; 6 Department of Ophthalmology, Nowon Eulji Medical Center, Eulji University School of Medicine, Seoul 01830, Republic of Korea

**Keywords:** 25-hydroxyvitamin D, intraocular pressure, glaucoma

## Abstract

Elevated intraocular pressure (EIOP) is the only major modifiable risk factor of glaucoma. While low serum vitamin D levels are considered a potential risk factor for glaucoma, there is conflicting evidence on the relationship between vitamin D and EIOP despite the possible linkage between vitamin D and intraocular pressure through oxidative stress and systemic inflammation. Therefore, the aim of this study is to verify the relationship between 25-hydroxyvitamin D [25(OH)D] and EIOP using data from 15,338 individuals who visited the health promotion center of an education hospital. The cubic spline curve revealed an inverse dose-dependent association between serum 25(OH)D level and EIOP. Using multiple logistic regression analysis, the fully adjusted odds ratio (OR) with 95% confidence interval (CI) for the EIOP of the serum 25(OH)D per increment was 0.97 (0.96–0.990). The fully adjusted ORs (95% CIs) for the EIOP of the 25(OH)D insufficiency and 25(OH)D sufficiency groups, compared to 25(OH)D deficiency group, were 0.72 (0.56–0.92) and 0.51 (0.34–0.78), respectively. The relationship remained significant in male and young age subgroups. In conclusion, the clinical assessment of intraocular pressure may prove helpful when treating patients with 25(OH)D deficiency, which may be a preventive strategy against the development of glaucoma.

## 1. Introduction

Glaucoma is a leading cause of irreversible vision impairment worldwide. According to a systematic review, it accounts for 8% of blindness and distance vision impairment, making it the fourth most common cause of blindness [1]. By 2020, there were approximately 76 million people who suffered from glaucoma [2]. In addition, there are expected to be 111.8 million people with glaucoma by 2040 [2]. Moreover, in 2013, it was estimated that the socioeconomic costs for glaucoma in Korea amounted to 3 trillion KRW [3].

Although the pathophysiology of glaucoma is not completely understood, oxidative stress and elevated intraocular pressure (EIOP) play a role in its development and progression. Oxidative stress may induce neuro-inflammation and the resistance to aqueous humor outflow is increased by hydrogen peroxide by inducing the degeneration of the trabecular meshwork [4]. Moreover, in patients with glaucoma, both the severity of defects on the visual field and intraocular pressure increase with an increasing amount of oxidative DNA damage affecting the trabecular meshwork [5]. Meanwhile, intraocular pressure can induce mechanical stress on the tissues of the optic nerve head, which contributes to the development and progression of glaucomatous optic neuropathy [6,7]. Thus, EIOP has been considered the primary and only modifiable risk factor for glaucomatous optic neuropathy [8]. Epidemiological evidence suggests that, in patients who did not undergo EIOP treatment, the proportion of patients with newly developed primary open-angle glaucoma over 5 years of follow-up ranged from 9.5% to 17.4% [9,10,11]. However, the significance of intraocular pressure measurement in the diagnosis of glaucoma has been changing because normal tension glaucoma is very common in Asian people, and especially in Korean people. Kim et al. [12] reported that the prevalence of patients with open-angle glaucoma with normal intraocular pressure was 2.7%, while that of patients with open-angle glaucoma with EIOP was 0.8%. Nevertheless, considering 23% of patients with open-angle glaucoma have EIOP, the early detection of EIOP is still one of the important factors for the prevention of glaucoma although several risk factors potentially contribute to the development of glaucoma [13]. Unfortunately, intraocular pressure measurement is not routinely performed in clinical practice except in ophthalmology clinics. Moreover, most patients with EIOP are asymptomatic, which makes it difficult for clinicians to detect EIOP unless the patient visits an ophthalmology clinic. Therefore, identifying clinical markers related to EIOP would be useful.

Vitamin D plays a role in reducing inflammation and angiogenesis. Vitamin D purportedly decreases the protein expression of toll-like receptor-2 and toll-like receptor-4, and decreases reactive oxygen species through an increase in cellular glutathione [14]. Although 25-hydroxyvitamin D3 [25(OH)D] can be synthesized from 7-dehydrocholesterol within keratinocytes in the human skin in response to ultraviolet B radiation [15], the prevalence of vitamin D deficiency is reportedly high. The prevalence of vitamin D deficiency is estimated to be 24% in the US and 40% in Europe [16]. Moreover, its prevalence in Korea has consistently increased from 51.8% in men and 68.2% in women in 2008 to 75.2% in men and 82.5% in women in 2014 [17]. Furthermore, its prevalence increases with age [18]. Interestingly, the intraocular pressure in women and older individuals decreases less than that in men and in younger age groups [19], which may denote an association between vitamin D deficiency and intraocular pressure. According to the current evidence, serum vitamin D levels are inversely related to glaucoma [20]. However, to our knowledge, the relationship between vitamin D levels and intraocular pressure has not been sufficiently verified. Focusing on the anti-inflammatory effects of vitamin D, we hypothesized that serum 25(OH)D levels are inversely related to EIOP. This study aimed to verify the associations of EIOP with serum 25(OH)D levels and 25(OH)D status using data from health examiners at a single center.

## 2. Materials and Methods

### 2.1. Study Population

**Figure 1:**
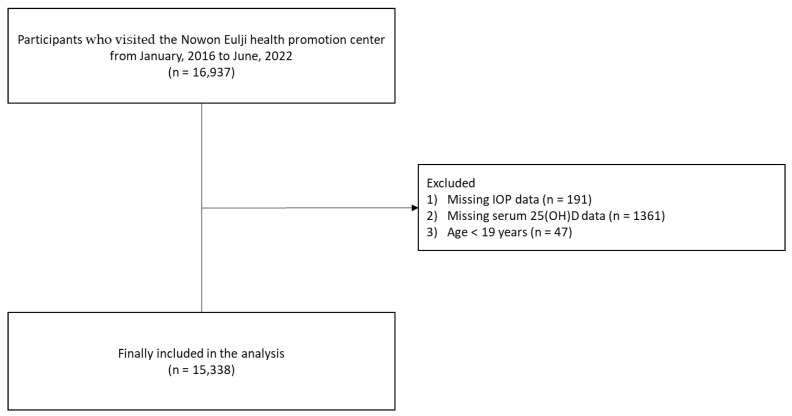
Flowchart of the study population selection. Abbreviations: intraocular pressure, intraocular pressure; 25(OH)D, 25-hydroxyvitamin D.

This retrospective, cross-sectional study used data from the health promotion center of an educational hospital. From January 2016 to June 2022, a total of 16,937 participants visited and received a health examination at the Nowon Eulji Health Promotion Center. The exclusion criteria were as follows: (1) those with missing intraocular pressure data (*n* = 191), (2) those with missing serum 25(OH)D data (*n* = 1361), and (3) those aged < 19 years (*n* = 47). We finally analyzed the data from 15,338 participants (Figure 1).

This study conformed to the ethical guidelines of the 1964 Declaration of Helsinki and its later amendments. Because it was a retrospective, cross-sectional study and researchers only accessed and analyzed the de-identified data, informed consent was waived. The Eulji University College of Medicine Ethics Committee approved the study protocol (IRB number: 2022-07-022).

### 2.2. Intraocular Pressure Measurement

Two well-trained paramedic assistants measured the intraocular pressure using a Topcon CT-80 non-contact tonometer (Topcon Medical Systems, Paramus, NJ, USA) without applying topical anesthetics. To minimize the effect of the diurnal fluctuation of intraocular pressure, measurements were taken in the morning from 08:00 AM to 11:00 AM. The average of two consecutive measurements was used to calculate the intraocular pressure. We defined EIOP as an intraocular pressure of 22 mmHg or more.

### 2.3. Serum 25(OH)D Measurement

Serum 25(OH)D levels were measured using a Chemistry XPT (Siemens Healthcare Diagnostics, Deerfield, MA, USA). In terms of these measurements, participants were classified into a group with 25(OH)D deficiency (serum 25(OH)D level less than 20 ng/mL), a group with 25(OH)D insufficiency (serum 25(OH)D level 20–29 ng/mL), or a group with 25(OH)D sufficiency (serum 25(OH)D level 30 ng/mL or more) [21].

### 2.4. Data Collection

The patients were divided into a young group (age < 65 years) and an elderly group (age ≥ 65 years). Due to a lack of information on menopausal status, we classified menopausal status as premenopausal status (age < 50 years) and postmenopausal status (age ≥ 50 years). Each participant’s height (m) and weight (kg) were measured to the nearest 0.001 m and 0.1 kg, respectively. Body mass index (BMI) was calculated as the waist circumference (cm) measured at the midpoint between the iliac crest and the lowest rib. After at least 30 mins of rest, systolic blood pressure (SBP, mmHg) and diastolic blood pressure (DBP, mm Hg) were measured in the sitting position.

Blood samples of each participant were collected from the anterior cubital vein after at least 12 h of fasting. Whole-blood hematocrit was analyzed using ADVIA 2120i (Siemens Healthcare Diagnostics, Deerfield, MA, USA). Using Chemistry XPT (Siemens Healthcare Diagnostics, Deerfield, MA, USA), fasting plasma glucose, serum total cholesterol, triglycerides, high-density lipoprotein cholesterol, low-density lipoprotein (LDL) cholesterol, and high-sensitivity C-reactive protein (hsCRP) levels were analyzed. 

Each participant recorded information about their smoking status, alcohol drinking status, and physical activity, which was confirmed through a face-to-face interview with a medical doctor. In terms of smoking status, participants were classified as non-smokers, ex-smokers, or current smokers. In terms of alcohol consumption status, they were classified as current drinkers and non-drinkers. Participants with ≥ 20 minutes of high-intensity physical activity ≥ 3 times a week or ≥ 30 minutes of moderate-intensity physical activity ≥ 5 times a week were defined as regular exercisers.

### 2.5. Statistical Analysis

All data are presented as the mean ± standard deviation for continuous variables and number (percentage, %) for categorical variables. Analysis of variance was used to compare the differences in continuous variables among groups. The chi-square test was used to compare differences in categorical variables among groups. 

We identified the dose–response relationship between serum 25(OH)D level and risk for EIOP using a cubic spline curve. Multiple logistic regression analysis was performed to estimate the odds ratio (OR) and 95% confidence interval (CI) for EIOP in the 25(OH)D deficiency and 25(OH)D insufficiency groups compared with the reference 25(OH)D sufficiency group. In Model 1, we adjusted for age, sex, and BMI. In Model 2, we adjusted the variables included in Model 1 in addition to smoking status, alcohol consumption status, and exercise status. In Model 3, we adjusted the variables included in Model 2 plus SBP, hematocrit, fasting plasma glucose, serum hsCRP, and serum LDL cholesterol levels. To display the results of subgroup analysis by sex and age group, forest plots were used.

All statistical analyses were conducted using the SPSS software version 25 (SPSS Inc., Chicago, IL, USA) and R (version 4.1.1; R Foundation for Statistical Computing, Vienna, Austria). All statistical tests were two-sided, and statistical significance was set at *p* < 0.05.

## 3. Results

Table 1 shows the clinical characteristics of the study population. The number of participants with 25(OH)D deficiency, 25(OH)D insufficiency, and 25(OH)D sufficiency was 9800, 3685, and 1853, respectively. The proportion of men, current smokers, and current drinkers were the lowest in people with 25(OH)D sufficiency compared with other groups. The mean values of BMI, serum LDL cholesterol level, and serum hsCRP level were the lowest in people with 25(OH)D sufficiency compared with other groups. There were no significant differences in the mean values of age, SBP, DBP, whole-blood hematocrit, and fasting plasma glucose levels among groups.

**Figure 2:**
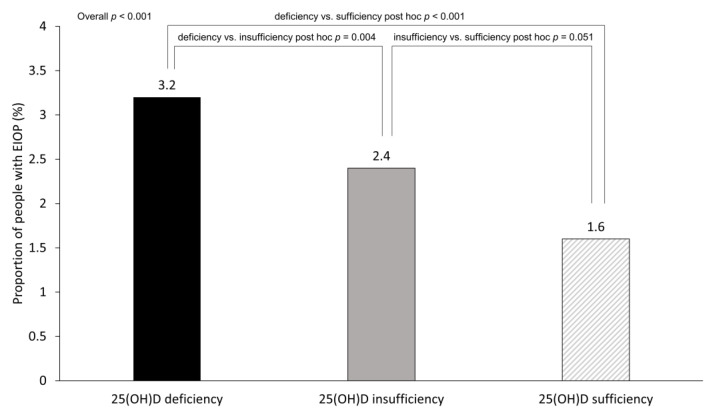
The proportion of people with EIOP according to 25(OH)D status. Chi-square tests were used to compare differences in the proportion of people with EIOP among groups. Pairwise comparison tests were completed. A *p*-value < 0.05 was considered statistically significant. Abbreviation: 25(OH)D, 25-hydroxyvitamin D; EIOP, elevated intraocular pressure.

Figure 2 shows the proportion of people with EIOP according to the 25(OH)D status. The proportion of people with EIOP was highest in the 25(OH)D deficiency group, followed by the 25(OH)D insufficiency and 25(OH)D sufficiency groups (3.2% in the 25(OH)D deficiency group, 2.4% in the 25(OH)D insufficiency group, and 1.6% in the 25(OH)D sufficiency group, overall *p* < 0.001). Pairwise comparisons revealed significant differences in the proportion of people with EIOP between the 25(OH)D deficiency group and the 25(OH)D insufficiency group (post hoc *p* = 0.004), and the 25(OH)D deficiency group and the 25(OH)D sufficiency group (post hoc *p* < 0.001). There was no significant difference in the proportion of people with EIOP between the 25(OH)D insufficiency group and the 25(OH)D sufficiency group (post hoc *p* = 0.051).

**Figure 3:**
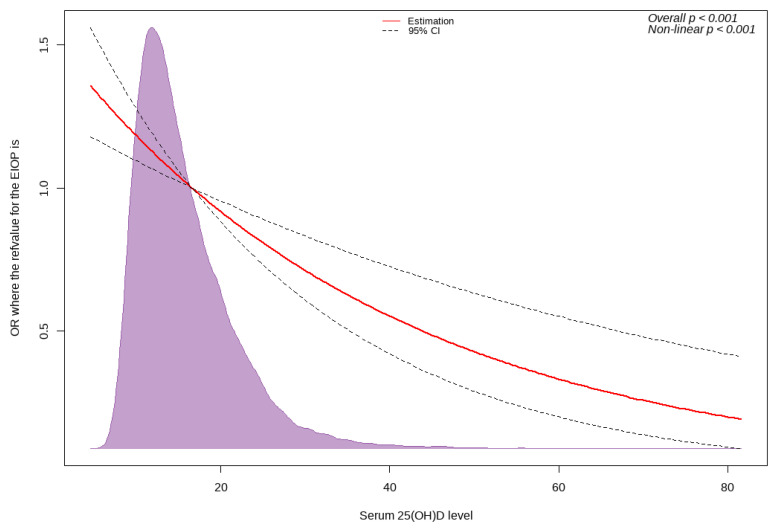
Cubic spline curve showing inverse dose–response relationship between serum 25(OH)D level and risk of EIOP. Abbreviations: OR, odds ratio; CI, confidence interval; 25(OH)D, 25-hydroxyvitamin D3; EIOP, elevated intraocular pressure.

A cubic spline curve revealed a significant inverse relationship between serum 25(OH)D levels and the OR for EIOP (Figure 3). As serum 25(OH)D level increased, the risk of EIOP decreased (overall *p* < 0.001).

Table 2 presents the results of the independent relationship between serum 25(OH)D levels and EIOP using multiple logistic regression analysis. The ORs and 95% CIs for EIOP in the 25(OH)D insufficiency and 25(OH)D sufficiency groups compared with the 25(OH)D deficiency group were 0.72 (0.57–0.92) and 0.47 (0.32–0.70), respectively. The relationship remained significant in adjustment Model 1, Model 2, and Model 3. The adjusted ORs and 95% CIs for EIOP in the 25(OH)D insufficiency and 25(OH)D sufficiency groups compared with the 25(OH)D deficiency group were 0.71 (0.56–0.91) and 0.56 (0.38–0.83) in Model 1, 0.71 (0.56–0.90) and 0.55 (0.37–0.82) in Model 2, and 0.72 (0.56–0.92) and 0.51 (0.34–0.78) in Model 3, respectively. The fully adjusted OR and 95% CI for EIOP of serum 25(OH)D level per increment was 0.97 (0.96–0.99) (Table S1 (Supplementary Materials)).

**Figure 4:**
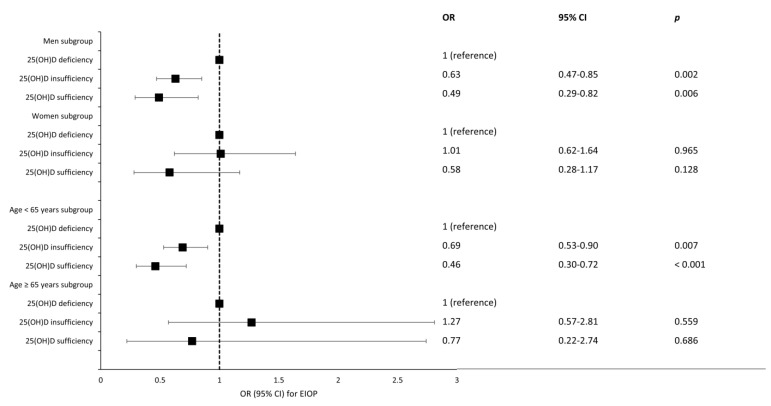
Forest plot showing the relationship between 25(OH)D status and EIOP in the sex subgroup and age subgroup. *p*-value < 0.05 considered as statistical significance. Abbreviation: 25(OH)D, 25-hydroxyvitamin D; EIOP, elevated intraocular pressure; OR, odds ratio; CI, confidence interval.

Figure 4 shows the results of the subgroup analyses by sex and age group. In the male subgroup, the fully adjusted ORs (95% CI) for EIOP in the 25(OH)D insufficiency and 25(OH)D sufficiency groups were 0.63 (0.47–0.85) and 0.49 (0.29–0.82), respectively. In the young age subgroup, the fully adjusted ORs (95% CI) for EIOP in the 25(OH)D insufficiency and 25(OH)D sufficiency groups were 0.69 (0.53–0.90) and 0.46 (0.30–0.72), respectively. In the female and elderly subgroups, the 25(OH)D status exhibited no significant relationship with EIOP. In the female subgroup, the fully adjusted ORs (95% CI) for EIOP in the 25(OH)D insufficiency and 25(OH)D sufficiency groups were 1.01 (0.62–1.64) and 0.58 (0.28–1.17), respectively. In the elderly subgroup, the fully adjusted ORs (95% CI) for EIOP in the 25(OH)D insufficiency and 25(OH)D sufficiency groups were 1.27 (0.57–2.81) and 0.77 (0.22–2.74), respectively.

## 4. Discussion

This cross-sectional study revealed a significant association of the 25(OH)D status with EIOP. The proportion of people with EIOP was highest in the 25(OH)D deficiency group, followed by the 25(OH)D insufficiency group and 25(OH)D sufficiency group. Serum 25(OH)D levels were inversely related to EIOP. Compared with that in people with 25(OH)D deficiency, those with 25(OH)D insufficiency and 25(OH)D sufficiency were at a lower risk of EIOP. Moreover, a significant relationship between 25(OH)D status and EIOP was observed in the male and young subgroups, which may imply that more attention is needed to evaluate intraocular pressure for men with 25(OH)D deficiency and young people with 25(OH)D deficiency.

The biological action of vitamin D is mediated by the vitamin D receptor (VDR), which acts as a heterodimer with the retinoid X receptor [22]. The VDR/retinoic X receptor heterodimeric complex interacts with specific DNA sequences that are related to the vitamin D response element. This can result in the suppression or activation of the transcription of genes that influence vitamin D function. An experimental study confirmed that 1α,25-dihydroxyvitamin D3 attenuates oxidative stress-induced damage in human trabecular meshwork cells by inhibiting the transforming growth factor beta-SMAD family member 3-VDR pathway [23]. Our results support those of the previous studies. However, in a case-control study, the administration of vitamin D3 did not affect the intraocular pressure [24]. Although the intraocular pressure decreased by 0.8 mmHg after 6 months of vitamin D supplementation (20,000 IU twice per week) in the intervention group, the change in the intervention group was not significantly different from that in the placebo group (*p* = 0.92, independent *t*-test). However, a limitation of the study was that factors that could affect vitamin D and/or intraocular pressure—dietary habits, physical activity, or medications such as duloxetine and corticosteroids— were not considered. Further, the small sample size consisting only of Caucasians was also a limitation of this study. A large sample size, different race, and adjustment for potential confounders such as physical activity and medical history could be the reasons for the different results from those of the previous study.

While the subgroup analysis of males revealed a significant relationship between 25(OH)D status and EIOP in this study, the subgroup analysis of females did not. The mean intraocular pressure in females was lower than that in males (15.3 ± 2.9 for females vs. 16.1 ± 2.9 for males, *p* < 0.001). Several studies have suggested a protective effect of estrogen against the development of glaucoma [25,26]. A 1–3 mmHg lower intraocular pressure is observed in premenopausal women than in postmenopausal women. Moreover, estrogen therapy in postmenopausal women decreases intraocular pressure from 0.5 to 3 mmHg. Furthermore, estrogen receptor β polymorphisms have a significant association with the risk of open-angle glaucoma [27]. Thus, we hypothesized that the protective effect of estrogen on intraocular pressure could outweigh that of 25(OH)D. To determine the possible confounding effect of estrogen, we further performed subgroup analysis by menopausal status. However, neither premenopausal nor postmenopausal women showed a significant association between 25(OH)D and EIOP although the risk of EIOP in the 25(OH)D sufficiency group tended to be lower than that in the 25(OH)D deficiency group (unadjusted OR = 0.29, 95% CI:0.07–1.22, *p* = 0.090) (Table S2). The possible interaction between 25(OH)D and sex hormones, such as estrogen, follicular stimulating hormone, or anti-Müllerian hormone, on EIOP should be considered in future studies.

Systemic inflammation is also associated with intra-orbital changes. In a cross-sectional study, serum C-reactive protein levels negatively correlated with choroidal and retinal thickness [28]. Additionally, Lee et al. [29] reported that the intraocular pressure significantly increases with increasing serum C-reactive protein tertiles, regardless of concordant metabolic syndrome. Alterations in mitochondrial energy metabolism coupled with a reduction in oxidant defenses that occur during vitamin D deficiency may act concurrently to increase reactive oxygen species formation and may be a universal driver of age-related diseases. Vitamin D deficiency contributes to the deregulation of calcium metabolism and redox cell-signaling pathways [30]. Vitamin D may play a major role in regulating the rate of aging, since normal levels of vitamin D are capable of maintaining the aging processes at low rates [31]. Moreover, a previous study suggested an association of vitamin D deficiency with oxidative stress, independent of sex and age [32]. Therefore, we expected 25(OH)D status to be associated with EIOP in the elderly group. However, a significant association was observed between 25(OH)D status and EIOP in the young age group but not in the elderly group. Elevated blood inflammatory marker levels are found in most older people [33]. Although 25(OH)D is suggested to have anti-inflammatory properties [14], the effects of 25(OH)D on intraocular pressure may be attenuated by chronic inflammation in elderly people. This may also be due to the relatively low number of samples in the elderly (*n* = 1173; 25(OH)D deficiency group (*n* = 747), 25(OH)D insufficiency group (*n* = 274), and 25(OH)D sufficiency group (*n* = 152)). Further well-designed clinical trials should be performed to confirm the effect of 25(OH)D on intraocular pressure, considering the mediating effect of systemic inflammation.

This study had several limitations. First, we could not identify a causal relationship between serum 25(OH)D levels and EIOP due to the cross-sectional study design. The role of 25(OH)D levels in the development of glaucoma should be confirmed with large-scale clinical trials. Secondly, information regarding diurnal changes in intraocular pressure, corneal thickness, angle structure, axial length of the eye, glaucomatous status, and whether participants received intraocular pressure-lowering treatment was lacking. Importantly, since angle structure has more established effects on intraocular pressure than 25(OH)D status, it is essential to identify the relationship between 25(OH)D status and EIOP considering angle structure in future studies. Finally, our results may not be applicable to populations of other ethnicities, because only Korean data were analyzed. Despite these limitations, this was the first study to verify the relationship between 25(OH)D levels and EIOP using a large quantity of population-based data.

## 5. Conclusions

Serum 25(OH)D levels are inversely associated with EIOP. In particular, people with 25(OH)D deficiency are at a higher risk of EIOP than people with 25(OH)D insufficiency or 25(OH)D sufficiency. Thus, it may be helpful for clinicians to check intraocular pressure when treating patients with 25(OH)D deficiency, as this may be a preventive strategy against glaucoma. More randomized controlled trials should be performed to confirm the causal relationship between 25(OH)D levels and glaucoma development, with consideration of the angle structure. Experimental studies are warranted to identify the association between 25(OH)D and intraocular pressure.

## Figures and Tables

**Table 1:** Clinical characteristics of the study population.

Variables	25(OH)D Deficiency	25(OH)D Insufficiency	25(OH)D Sufficiency	*p*
	(*n* = 9800)	(*n* = 3685)	(*n* = 1853)	
Men, *n* (%)	5409 (55.2%)	2216 (60.1%)	830 (44.8%)	<0.001
Age, years	46.3 ± 12.2	46.1 ± 12.3	46.2 ± 12.7	0.492
BMI, kg/m^2^	24.1 ± 3.7	24.1 ± 3.3	23.1 ± 3.2	<0.001
SBP, mmHg	120.4 ± 14.2	121.3 ± 13.8	120.5 ± 14.7	0.144
DBP, mmHg	73.2 ± 10.4	73.6 ± 10.2	73.0 ± 10.1	0.684
Smoking status, *n* (%)				<0.001
Non-smoker	7060 (72.2%)	2668 (72.5%)	1404 (76.2%)	
Ex-smoker	884 (9.0%)	404 (11.0%)	207 (11.2%)	
Current smoker	1830 (18.7%)	606 (16.5%)	231 (12.5%)	
Current drinker, *n* (%)	5349 (54.7%)	2004 (54.5%)	940 (51.0%)	0.013
Regular exerciser, *n* (%)	1650 (16.9%)	797 (21.7%)	473 (25.7%)	<0.001
Hematocrit, %	43.1 ± 4.2	43.3 ± 3.9	42.8 ± 3.8	0.263
Glucose, mg/dL	91.6 ± 22.4	91.8 ± 18.9	92.0 ± 17.9	0.348
LDL cholesterol, mg/dL	120.9 ± 34.8	121.5 ± 35.6	118.1 ± 36.0	0.021
hsCRP, mg/dL	0.13 ± 0.28	0.13 ± 0.30	0.11 ± 0.21	0.046

For continuous variables, an analysis of variance was used. For categorical variables, a chi-square test was used. A *p*-value < 0.05 was considered statistically significant. Abbreviations: 25(OH)D, 25-hydroxyvitamin D; BMI, body mass index; SBP, systolic blood pressure; DBP, diastolic blood pressure; LDL, low-density lipoprotein; hsCRP, high-sensitivity C-reactive protein.

**Table 2:** Relationship between 25(OH)D status and EIOP.

	25(OH)D Deficiency	25(OH)D Insufficiency		25(OH)D Sufficiency	
EIOP	OR	OR (95% CI)	*p*-Value	OR (95% CI)	*p*
Unadjusted	1 (reference)	0.72 (0.57–0.92)	0.008	0.47 (0.32–0.70)	<0.001
Model 1	1 (reference)	0.71 (0.56–0.91)	0.006	0.56 (0.38–0.83)	0.003
Model 2	1 (reference)	0.71 (0.56–0.90)	0.005	0.55 (0.37–0.82)	0.003
Model 3	1 (reference)	0.72 (0.56–0.92)	0.009	0.51 (0.34–0.78)	0.002

Model 1: adjusted for age, sex, and BMI. Model 2: adjusted for variables included in Model 1 plus smoking status, currently drinking status, and exercise status. Model 3: adjusted for variables included in Model 2 plus SBP, hematocrit, fasting plasma glucose, serum hsCRP, and serum LDL cholesterol levels. Abbreviations: 25(OH)D, 25-hydroxyvitamin D; EIOP, elevated intraocular pressure; OR, odds ratio; CI, confidence interval; BMI, body mass index; SBP, systolic blood pressure; hsCRP, high-sensitivity C-reactive protein; LDL, low-density lipoprotein.

## Data Availability

Not applicable.

## Supplementary Materials

The following supporting information can be downloaded at: https://www.mdpi.com/article/10.3390/nu15020423/s1, Table S1: Inverse relationship between serum 25(OH)D level and risk of elevated intraocular pressure; Table S2: Relationship between 25(OH)D status and risk of elevated intraocular pressure according to the menopausal status.

## References

[1] Flaxman S.R., Bourne R.R.A., Resnikoff S., Ackland P., Braithwaite T., Cicinelli M.V., Das A., Jonas J.B., Keeffe J., Kempen J.H. (2017). Global causes of blindness and distance vision impairment 1990–2020: A systematic review and meta-analysis. Lancet Glob. Health.

[2] Allison K., Patel D., Alabi O. (2020). Epidemiology of Glaucoma: The Past, Present, and Predictions for the Future. Cureus.

[3] Ahn Y., Jee D. (2018). Socioeconomic Costs of Glaucoma in Korea. J. Korean Ophthalmol. Society.

[4] Izzotti A., Bagnis A., Saccà S.C. (2006). The role of oxidative stress in glaucoma. Mutat. Res..

[5] Baudouin C., Kolko M., Melik-Parsadaniantz S., Messmer E.M. (2021). Inflammation in Glaucoma: From the back to the front of the eye, and beyond. Prog. Retin. Eye Res..

[6] Sigal I.A., Ethier C.R. (2009). Biomechanics of the optic nerve head. Exp. Eye Res..

[7] Strouthidis N.G., Girard M.J. (2013). Altering the way the optic nerve head responds to intraocular pressure-a potential approach to glaucoma therapy. Curr. Opin. Pharm..

[8] Park S.A., Komáromy A.M. (2021). Biomechanics of the optic nerve head and sclera in canine glaucoma: A brief review. Vet. Ophthalmol..

[9] Gordon M.O., Beiser J.A., Brandt J.D., Heuer D.K., Higginbotham E.J., Johnson C.A., Keltner J.L., Keltner J.L., Miller J.P., Parrish II R.K. (2002). The Ocular Hypertension Treatment Study: Baseline factors that predict the onset of primary open-angle glaucoma. Arch. Ophthalmol..

[10] Gordon M.O., Kass M.A. (2018). What We Have Learned From the Ocular Hypertension Treatment Study. Am. J. Ophthalmol..

[11] Thomas R., George R., Parikh R., Muliyil J., Jacob A. (2003). Five year risk of progression of primary angle closure suspects to primary angle closure: A population based study. Br. J. Ophthalmol..

[12] Kim C.S., Seong G.J., Lee N.H., Song K.C. (2011). Prevalence of primary open-angle glaucoma in central South Korea the Namil study. Ophthalmology.

[13] McMonnies C.W. (2017). Glaucoma history and risk factors. J. Optom..

[14] Calton E.K., Keane K.N., Newsholme P., Soares M.J. (2015). The Impact of Vitamin D Levels on Inflammatory Status: A Systematic Review of Immune Cell Studies. PLoS ONE.

[15] Morris H., Anderson P. (2010). Autocrine and Paracrine Actions of Vitamin D. Clin. Biochem. Rev./Aust. Assoc. Clin. Biochem..

[16] Amrein K., Scherkl M., Hoffmann M., Neuwersch-Sommeregger S., Köstenberger M., Tmava Berisha A., Martucci G., Pilz S., Malle O. (2020). Vitamin D deficiency 2.0: An update on the current status worldwide. Eur. J. Clin. Nutr..

[17] Park J.-H., Hong I.Y., Chung J.W., Choi H.S. (2018). Vitamin D status in South Korean population: Seven-year trend from the KNHANES. Medicine.

[18] Song H.-R., Kweon S.-S., Choi J.-S., Rhee J.-A., Lee Y.-H., Nam H.-S., Jeong S.-K., Park K.-S., Ryu S.-Y., Choi S.-W. (2014). High Prevalence of Vitamin D Deficiency in Adults Aged 50 Years and Older in Gwangju, Korea: The Dong-gu Study. J Korean Med. Sci..

[19] Baek S.U., Kee C., Suh W. (2015). Longitudinal analysis of age-related changes in intraocular pressure in South Korea. Eye.

[20] Kim H.T., Kim J.M., Kim J.H., Lee M.Y., Won Y.S., Lee J.Y., Park K.H. (2016). The Relationship between Vitamin D and Glaucoma: A Kangbuk Samsung Health Study. Korean J. Ophthalmol..

[21] Holick M.F., Binkley N.C., Bischoff-Ferrari H.A., Gordon C.M., Hanley D.A., Heaney R.P., Murad M.H., Weaver C.M. (2011). Evaluation, treatment, and prevention of vitamin D deficiency: An Endocrine Society clinical practice guideline. J. Clin. Endocrinol. Metab..

[22] Caban M., Lewandowska U. (2022). Vitamin D, the Vitamin D Receptor, Calcitriol Analogues and Their Link with Ocular Diseases. Nutrients.

[23] Lv Y., Han X., Yao Q., Zhang K., Zheng L., Hong W., Xing X. (2019). 1α,25-dihydroxyvitamin D3 attenuates oxidative stress-induced damage in human trabecular meshwork cells by inhibiting TGFβ-SMAD3-VDR pathway. Biochem. Biophys. Res. Commun..

[24] Krefting E.A., Jorde R., Christoffersen T., Grimnes G. (2014). Vitamin D and intraocular pressure--results from a case-control and an intervention study. Acta Ophthalmol..

[25] Dewundara S.S., Wiggs J.L., Sullivan D.A., Pasquale L.R. (2016). Is Estrogen a Therapeutic Target for Glaucoma?. Semin. Ophthalmol..

[26] Feola A.J., Sherwood J.M., Pardue M.T., Overby D.R., Ethier C.R. (2020). Age and Menopause Effects on Ocular Compliance and Aqueous Outflow. Investig. Ophthalmol. Vis. Sci..

[27] Ulhaq Z.S. (2020). The association of estrogen-signaling pathways and susceptibility to open-angle glaucoma. Beni-Suef Univ. J. Basic Appl. Sci..

[28] Fang D., Li Q., Yan K., Xu S., Jiang J., Che X., Zhang Y., Qian Y., Wang Z. (2021). Retinal and Choroidal Thickness in relation to C-Reactive Protein on Swept-Source Optical Coherence Tomography. J. Immunol. Res..

[29] Lee I.T., Wang J.-S., Fu C.-P., Chang C.-J., Lee W.-J., Lin S.-Y., Sheu W.H.-H. (2017). The synergistic effect of inflammation and metabolic syndrome on intraocular pressure: A cross-sectional study. Medicine.

[30] Berridge M.J. (2016). Vitamin D, reactive oxygen species and calcium signalling in ageing and disease. Philos. Trans. R. Soc. B Biol. Sci..

[31] Boaventura B.C.B., Cembranel F., Preedy V.R., Patel V.B. (2020). Chapter 35—Protective effect of vitamin D on oxidative stress in elderly people. Aging.

[32] Câmara A.B., Brandão I.A. (2021). The relationship between vitamin D deficiency and oxidative stress can be independent of age and gender. Int. J. Vitam. Nutr. Res..

[33] Ferrucci L., Fabbri E. (2018). Inflammageing: Chronic inflammation in ageing, cardiovascular disease, and frailty. Nat. Rev. Cardiol..

