# Supplementary material for: Inverse Relationship between Serum 25-Hydroxyvitamin D and Elevated Intraocular Pressure
Source: Nutrients. 2023 Jan 13;15(2):423. doi: 10.3390/nu15020423 (PMC9866375; doi:10.3390/nu15020423)
Supplement: Supplementary file 1 [file nutrients-15-00423-s001.zip › nutrients-2115687-supplementary.pdf]

Table S1. Relationship between serum 25(OH)D level and EIOP.

EIOP	25(OH)D level per increment<br>OR (95% CI)	*p*
Unadjusted	0.98 (0.96–0.99)	< 0.001
Model 1	0.98 (0.96–0.99)	< 0.001
Model 2	0.98 (0.96–0.99)	< 0.001
Model 3	0.97 (0.96–0.99)	< 0.001

Model 1: adjusted for age, sex, and BMI.

Model 2: adjusted for variables included in Model 1 plus smoking status, currently drinking status, and exercise status.

Model 3: adjusted for variables included in Model 2 plus SBP, hematocrit, fasting plasma glucose, serum LDL cholesterol, and serum hsCRP levels.

Abbreviations: 25(OH)D, 25-hydroxyvitamin D; EIOP, elevated intraocular pressure; OR, odds ratio; CI, confidence interval; BMI, body mass index; SBP, systolic blood pressure; LDL, low-density lipoprotein; hsCRP, high-sensitivity C-reactive protein.

Table S2. Relationship between 25(OH)D status and EIOP by menopausal status.

	25(OH)D deficiency	25(OH)D insufficiency		25(OH)D sufficiency	
EIOP	OR	OR (95% CI)	*p*	OR (95% CI)	*p*
Premenopause					
Unadjusted	1 (reference)	0.80 (0.43–1.49)	0.482	0.75 (0.35–1.60)	0.461
Adjusted*	1 (reference)	0.85 (0.44–1.63)	0.620	0.74 (0.33–1.67)	0.467
Postmenopause					
Unadjusted	1 (reference)	1.35 (0.68–2.69)	0.390	0.29 (0.07–1.22)	0.090
Adjusted*	1 (reference)	1.34 (0.65–2.77)	0.435	0.32 (0.08–1.40)	0.132

*adjusted for age, SBP, hematocrit, fasting plasma glucose, serum LDL cholesterol, and serum hsCRP levels.

Abbreviations: 25(OH)D, 25-hydroxyvitamin D; EIOP, elevated intraocular pressure; OR, odds ratio; CI, confidence interval; BMI, body mass index; SBP, systolic blood pressure; LDL, low-density lipoprotein; hsCRP, high-sensitivity C-reactive protein.
